# Optilume Drug-Coated Balloon Dilation for Male Sphincteric (Membranous) Urethral Strictures: 53 Consecutive Cases

**DOI:** 10.3390/jcm14238369

**Published:** 2025-11-25

**Authors:** Lukas Andrius Jelisejevas, Gennadi Tulchiner, Peter Rehder

**Affiliations:** Department of Urology, Medical University Innsbruck, 6020 Innsbruck, Tyrol, Austria; gennadi.tulchiner@i-med.ac.at (G.T.); peter.rehder@i-med.ac.at (P.R.)

**Keywords:** urethral stricture, urinary retention, paclitaxel, urinary bladder neck obstruction, urinary catheterisation, urethral catheters, urinary diversion, lower urinary tract symptoms, cystoscopy

## Abstract

**Background/Objectives:** Reconstruction of membranous urethral strictures poses significant surgical challenges, including risks of urinary incontinence and erectile dysfunction. Optilume drug-coated balloon dilation (DCBD) is a minimally invasive treatment for short, recurrent bulbar urethral strictures, but its application in strictures involving the sphincteric urethra remains controversial. This study aims to evaluate the safety, efficacy, and impact on continence of DCBD in membranous urethral strictures involving the male sphincter. **Methods:** A retrospective analysis was conducted on 53 consecutive patients with urethral strictures involving the sphincteric urethra, treated with Optilume DCBD between June 2021 and June 2025 at a tertiary center. After preoperative imaging, dilation to 20 Fr, then DCBD (30 Fr, 10 bar, 10 min) were performed. We assessed anatomical success (≥18 Fr as per cystoscopy/calibration), freedom from re-intervention, and continence status. Patients with neurological conditions or urinary infections were excluded. **Results:** The cohort included 35 membranous urethral strictures and 18 vesicourethral anastomosis stenoses that extended into the sphincter. The median follow-up was 13.3 months. At last follow-up, 66.6% and 65.6% of patients in both groups were free from recurrence and re-intervention with satisfactory voiding. No de novo incontinence was observed; two patients with prior post-prostatectomy incontinence remained incontinent. The median age was 68 years; median prior interventions were 2.5, and median stricture length was 3 cm. **Conclusions**: Optilume DCBD appears to be a safe and effective option for membranous urethral strictures involving the sphincter, without inducing de novo incontinence. Although not a replacement for reconstruction, it offers a minimally invasive alternative for selected patients.

## 1. Introduction

Reconstruction of membranous urethral strictures poses significant surgical challenges. Several surgical techniques have been described, including ventral or dorsal onlay graft urethroplasty and intrasphincteric bulboprostatic anastomosis [1,2]. Traditionally, endoscopic treatments have been preferred over reconstructive procedures due to fear of complications, complex anatomy and functional significance of the region [3]. Recently, endoscopic buccal urethroplasty was proposed and demonstrated promising results [4]. Despite these advancements, the risk of complications remains a concern, with postoperative urinary incontinence rates reported to be as high as 35.7% [3,5].

Paclitaxel is a well-established antimitotic agent that inhibits fibroblast proliferation by stabilizing microtubules and preventing cell division, reducing scar tissue formation after dilation [6]. The rationale of its local delivery via a drug-coated balloon is to help maintain urethral patency by limiting restenosis through targeted antiproliferative and antifibrotic effects. Paclitaxel-coated devices have demonstrated a strong safety record across multiple medical applications, including their extensive use in the endovascular treatment of coronary artery disease and peripheral arterial disease [6,7].

Optilume drug-coated balloon (Laborie, Plymouth, MN, USA) has been introduced in December 2021. It combines mechanical dilation with the local delivery of paclitaxel. Drug-coated balloon dilation (DCBD) has demonstrated high efficacy in the treatment of short bulbar urethral strictures and has proven superior to standard endoscopic therapy in a randomized controlled trial [8]. However, its application in strictures involving the membranous (sphincteric) urethra remains uncertain [9,10,11]. This study aims to evaluate the safety, efficacy, and impact on continence of Optilume DCBD in membranous urethral strictures involving the male sphincter.

## 2. Materials and Methods

### 2.1. Study Population

We conducted a retrospective review of patients who received Optilume DCBD for urethral strictures involving membranous urethra at a single tertiary care center. These strictures involved the membranous (sphincteric) urethra directly, or the sphincter was involved as part of vesicourethral anastomotic stenosis (VUAS). During the study period, 53 patients underwent DCBD and were prospectively followed in accordance with our study protocol. Inclusion criteria consisted of male patients aged 18 or older with a history of urethral strictures that had been previously treated. Diagnosis was established based on the inability to pass a urethral catheter, urethrography findings, or endoscopic assessment. Patients with urinary tract infections or neurological conditions were excluded. Prior to treatment, written informed consent was obtained, during which patients were also counseled about the possibility of open surgical reconstruction. All patients opted for an endoscopic approach over reconstructive surgery. All procedures were performed by experienced reconstructive urologists specialized in urethral reconstruction and minimally invasive techniques. This study received approval from the institutional review board (study number: 1101/2022).

### 2.2. Surgical Procedure and Follow-Up

**Figure 1:**
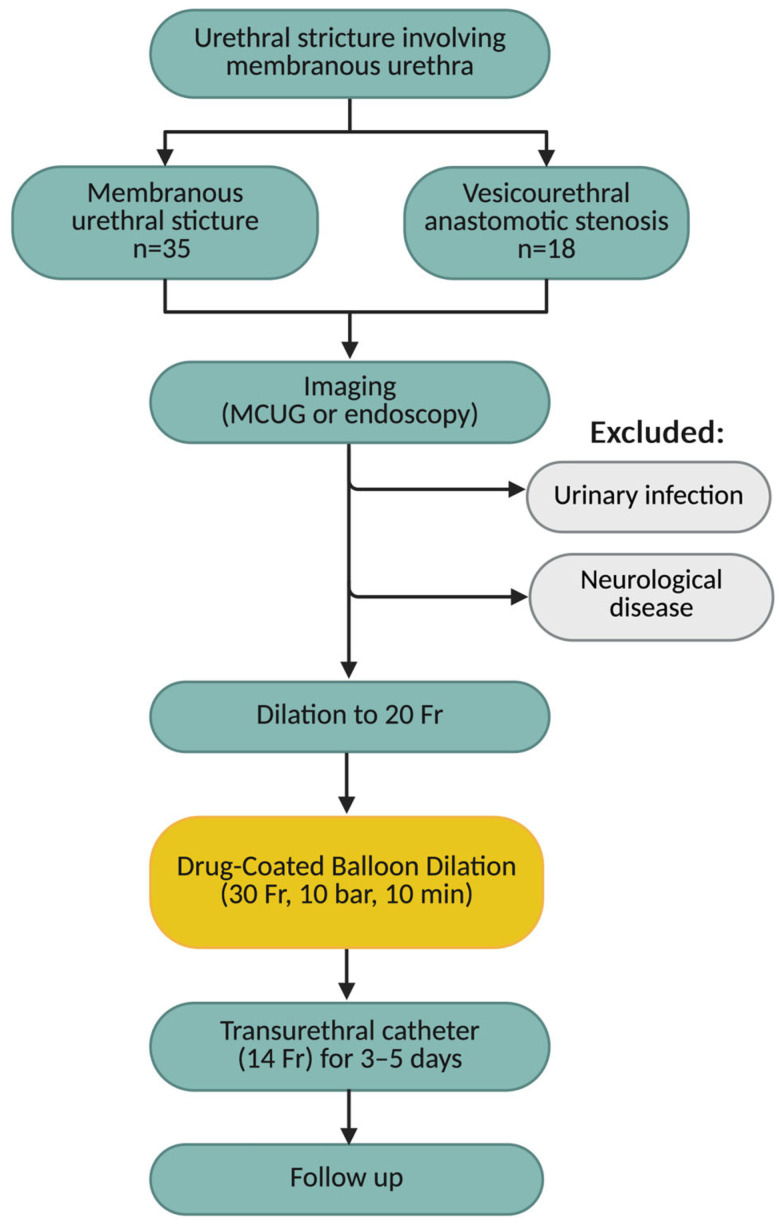
Flowchart of the study protocol. MCUG = micturating cystourethrogram.

The procedure was carried out with the patient in the lithotomy position. It was performed under either local or general anaesthesia, based on patient preference. For local anesthesia, intraurethral 2% lidocaine hydrochloride gel was applied 10 min prior to the procedure, with no premedication administered. Urethrocystoscopy was performed to assess the stricture and exclude other pathologies. Under cystoscopic guidance, a 0.035″ hydrophilic guidewire was passed through the urethra. The affected segment was then pre-dilated to 20 Fr using hydrophilic urethral dilators. The dilation device was filled with 20 mL of sterile saline. The drug-coated balloon (DCB) was positioned over the guidewire at the stricture location under direct endoscopic visual control. A 5 cm, 30 Fr balloon was placed so as to ensure at least 0.5 cm of overlap both proximally and distally to the stricture. The balloon was inflated to the recommended pressure of 10 bar, maintained for 10 min before deflation and removal. A 14 Fr Foley catheter was then inserted and kept in place for 3 to 5 days. Patients were advised to use barrier contraception for 30 days post-treatment to prevent exposure of sexual partners to paclitaxel. Men with partners of childbearing potential were recommended to use barrier contraception for at least 12 months. Follow-up evaluations were scheduled at 30 days after the procedure and subsequently at 3-month intervals in our outpatient clinic (see Figure 1).

### 2.3. Study Endpoint and Statistical Analysis

The primary endpoint of the study was anatomical success (defined as urethral calibration or cystoscopy confirming a lumen of ≥18 Fr) and freedom from reintervention. Change in continence status was assessed as a secondary endpoint. Reintervention was defined as any subsequent treatment for urethral strictures following the DCBD procedure. Statistical analyses were performed using IBM SPSS Version 30. Categorical variables were compared using the chi-squared test. Continuous variables were summarized as either mean ± standard deviation or median with interquartile range (IQR), depending on their distribution, which was assessed using the Kolmogorov–Smirnov test. For comparison of continuous variables, the Mann–Whitney U test or Student’s t-test was applied as appropriate. Variables considered in the analysis included stricture length, etiology, number of prior treatments, American Society of Anesthesiologists (ASA) class, diabetes mellitus, and history of pelvic radiation therapy.

## 3. Results

Between June 2021 and June 2025, a total of 53 consecutive male patients underwent Optilume DCBD for urethral strictures involving the sphincteric (membranous) urethra. Patient demographics and baseline characteristics are summarized in Table 1. Prior interventions included urethral dilation (including self-dilation), direct vision internal urethrotomy (DVIU), open urethroplasty, and anastomotic incision for VUAS.

**Figure 2:**
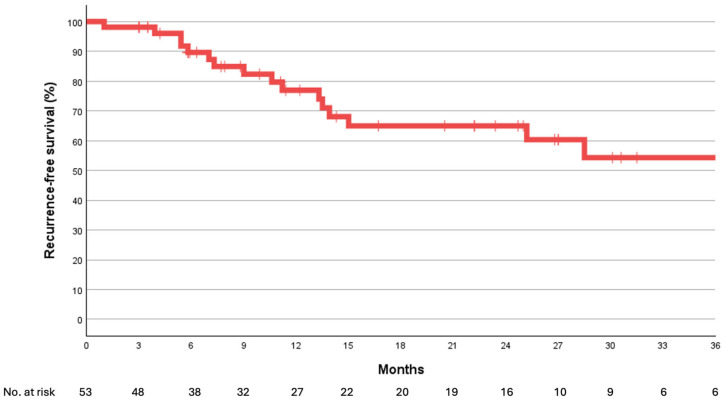
Kaplan–Meier curve illustrating freedom from recurrence following Optilume DCBD in 53 patients with urethral strictures involving the membranous (sphincteric) urethra.

**Figure 3:**
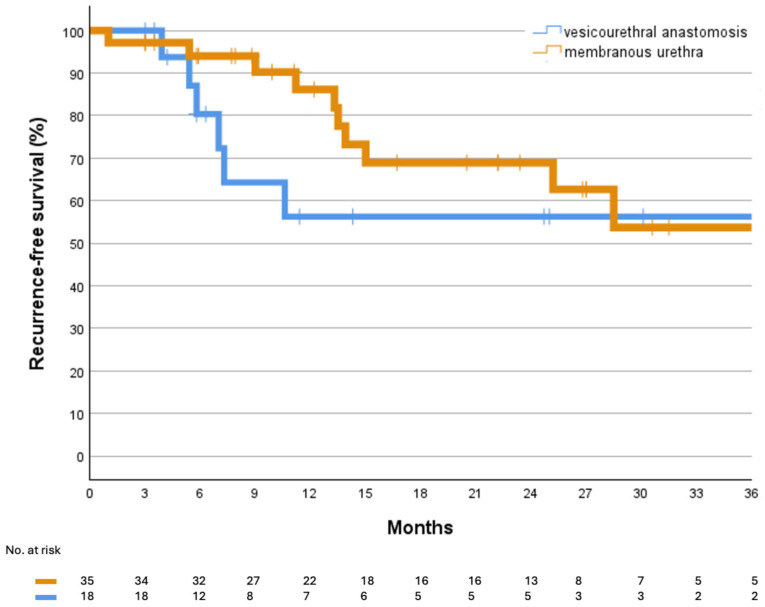
Kaplan–Meier curve comparing freedom from recurrence between patients with vesicourethral anastomosis stenosis (VUAS) and those with membranous urethral strictures following Optilume DCBD.

Of the 53 consecutive patients, 35 had strictures involving the membranous urethra, while 18 presented with VUAS involving the sphincteric segment. The median follow-up duration was 13.3 months [IQR: 7.0–27.4]. At the last follow-up, 66.6% (12/18) of patients with VUAS and 65.6% (24/35) of those with membranous urethral strictures remained free from stricture recurrence and repeat intervention, with satisfactorily voiding outcomes. Kaplan–Meier survival curves illustrating recurrence-free survival are shown in Figure 2 and Figure 3. Based on the Patient Global Impression of Improvement (PGI-I), all patients reported feeling “very much better” following the intervention. The median patient age was 68 years [IQR: 55–76], with a median of 2.5 prior interventions [IQR: 1–7.25] for urethral strictures. The median stricture length was 3 cm [IQR: 2–4]. On univariate analysis, prior pelvic irradiation (*p* = 0.047), higher ASA class (*p* = 0.028), and diabetes mellitus (*p* = 0.047) were significantly associated with treatment failure. No statistically significant differences were found between the VUAS and membranous stricture groups in terms of recurrence or continence outcomes. No cases of de novo urinary incontinence were observed following DCBD. Two complications (Clavien-Dindo Grade I) were noted: one case of urethral pain (1.9%) and one case of self-limiting urethral bleeding (1.9%). No changes in erectile function were reported after treatment.

## 4. Discussion

### 4.1. Current Treatment Strategies for Membranous Urethral Strictures and VUAS

Membranous urethral stricture typically arises from pelvic trauma, iatrogenic injury (e.g., TURP, catheterization), or radiation therapy [12]. Iatrogenic causes are most common in older adults, while trauma is more frequent in younger patients [13]. Data regarding the surgical management of true membranous urethral strictures remain limited, partly due to the ambiguous application of the term ‘membranous,’ which often includes proximal bulbar urethral strictures that may differ in both anatomy and pathology. Notably, even highly experienced urethral surgeons have observed that treatment of true membranous urethral strictures generally favors minimally invasive endoscopic techniques [14]. Kulkarni et al. reported treating such strictures endoscopically, with all patients subsequently requiring intermittent self-catheterization [14]. This underscores the complexity and unique challenges associated with the management of true membranous urethral strictures.

VUAS typically occurs after radical prostatectomy, with or without adjuvant radiation therapy [15]. Known risk factors include adjuvant radiation, high BMI, urine leak, blood transfusion, and non-nerve-sparing surgical techniques, as reported by Britton et al. [16]. Importantly, the development of VUAS is independently associated with an increased risk of post-prostatectomy urinary incontinence [16]. Endoscopic management—including dilation, incision, or resection—remains the first-line treatment; however, success rates are variable, and recurrence is common, especially after multiple procedures or in the context of prior radiation therapy [17]. Open or robotic urethroplasty is generally reserved for refractory cases. Among these, dorsal onlay buccal mucosa graft (BMG) urethroplasty has demonstrated favorable long-term patency [15]. However, the data on continence outcomes are conflicting. Some studies report that up to 59% of patients may experience new or worsened stress urinary incontinence after robotic VUAS repair [18]. Ultimately, many patients require the placement of an artificial urinary sphincter (AUS) to achieve continence [17,18].

### 4.2. Outcomes and Predictors of Recurrence and Continence

In present study, the recurrence rates observed in both VUAS and membranous urethral stricture groups were comparable, suggesting that DCBD may be equally applicable for both populations, with a median follow-up of 13.3 months and a recurrence-free rate of approximately 66%. Factors such as prior pelvic irradiation, higher ASA classification, and diabetes were associated with treatment failure, aligning with known predictors of poor outcomes in urethral stricture management [19,20]. These variables should be considered when selecting candidates for DCBD.

In this cohort, 19 patients (35.9%) were incontinent at baseline, reflecting previous surgical interventions such as radical prostatectomy. No de novo urinary incontinence was observed following DCBD. However, two patients with a history of stress urinary incontinence after radical prostatectomy had experienced temporary resolution of symptoms due to obstruction caused by the VUAS. Following successful dilation and restoration of urethral patency, their pre-existing incontinence re-emerged. These cases were therefore not classified as de novo incontinence, but rather as unmasking of previously existing incontinence. This distinction is critical in counseling patients and interpreting continence outcomes.

### 4.3. Clinical Role and Advantages of DCBD

While DCBD is not intended to replace reconstructive surgery, it provides a less invasive alternative for patients who are poor surgical candidates, prefer minimally invasive procedures or want to postpone definitive surgery. Its short procedural time, minimal anesthesia requirements, and rapid recovery make it attractive for elderly or comorbid populations.

### 4.4. Study Limitations

Limitations of this study include its retrospective design, relatively small sample size, and limited follow-up duration (median 13.3 months), which may be insufficient to fully assess long-term patency and recurrence. The absence of a control group prevents direct comparison with other treatment modalities. Additionally, since all patients were treated at a specialized reconstructive center, the generalizability of the results may be limited. Functional assessment was restricted to patient-reported outcomes using the PGI-I scale, as consistent and reliable uroflowmetry measurements were difficult to obtain and therefore excluded. While no cases of erectile dysfunction were observed following DCBD, we acknowledge that erectile function was not evaluated using validated instruments, which limits the strength of this observation. Consideration should also be given to emerging adjunctive strategies, including immuno-modulatory and regenerative approaches for managing secondary complications such as erectile dysfunction [21]. Prospective studies with larger cohorts and longer follow-up (≥24 months) are necessary to validate these findings and better define the role of DCBD in managing membranous urethral strictures.

## 5. Conclusions

Optilume DCBD demonstrates promising safety and efficacy in the management of membranous urethral strictures and VUAS involving the sphincter, without evidence of de novo urinary incontinence. While not intended to replace reconstruction, it may offer a useful minimally invasive option for selected patients for whom surgical reconstruction is less desirable.

## Figures and Tables

**Table 1:** Descriptive characteristics for 53 consecutive patients treated with Optilume DCBD for urethral strictures involving the membranous (sphincteric) urethra.

Parameter	Result
Age (years)	68 ± 15 [IQR: 55–76]
Body mass index (kg/m^2^)	26.2 ± 4.99 [IQR: 23.5–30.3]
Prior interventions	2.5 [IQR: 1–7.25]
PVR (baseline) (mL)	70 [IQR: 0–257.5]
PVR (postoperative) (mL)	0 [IQR: 0–11]
Follow-up (months)	13.3 ± [IQR: 7.0–27.4]
Stricture length (cm)	3 [IQR: 2–4]
Ever smoker	
Yes	13 (24.5%)
No	40 (75.5%)
Diabetes	
Yes	11 (20.75%)
No	42 (79.25%)
Irradiation	
Yes	17 (32%)
No	36 (68%)
Etiology	
Iatrogenic	37 (69.8%)
Congenital	6 (11.3%)
Idiopathic	5 (9.4%)
Traumatic	4 (7.6%)
Lichen sclerosus	1 (1.9%)
Stricture location	
Membranous urethra	35 (66%)
Vesicourethral anastomosis	18 (34%)
Complications (Clavien–Dindo)	
None	51 (96.2%)
Grade I	2 (3.8%)
ASA class	
ASA I	8 (15%)
ASA II	33 (62.3%)
ASA III	11 (20.8%)
ASA IV	1 (1.9%)
Preoperative incontinence status	
Patients with urinary incontinence	19 (35.9%)
Patients without urinary incontinence	34 (64.1%)
Postoperative incontinence status	
Patients with urinary incontinence	21 (39.6%)
Patients without urinary incontinence	32 (60.4%)
De novo incontinence	0 (0%)

## Data Availability

The original contributions presented in this study are included in the article. Further inquiries can be directed to the corresponding author.

## Abbreviations

ASA	American Society of Anesthesiologists
AUS	Artificial urinary sphincter
BMG	Buccal mucosa graft
BMI	Body mass index
DCB	Drug-coated balloon
DCBD	Drug-coated balloon dilation
DVIU	Direct vision internal urethrotomy
IQR	Interquartile range
MCUG	Micturating Cystourethrogram
PGI-I	Patient Global Impression of Improvement
PVR	Post-void residual
TURP	Transurethral resection of the prostate
VUA	Vesicourethral anastomosis
VUAS	Vesicourethral anastomotic stenosis
